# Kinetics and Adsorption Isotherms of Amine-Functionalized Magnesium Ferrite Produced Using Sol-Gel Method for Treatment of Heavy Metals in Wastewater

**DOI:** 10.3390/ma15114009

**Published:** 2022-06-05

**Authors:** Muhammad Irfan, Fareeda Zaheer, Humaira Hussain, Muhammad Yasin Naz, Shazia Shukrullah, Stanislaw Legutko, Mater H. Mahnashi, Mabkhoot A. Alsaiari, Abdulnour Ali Jazem Ghanim, Saifur Rahman, Omar Alshorman, Fahad Salem Alkahtani, Mohammad K. A. Khan, Izabela Kruszelnicka, Dobrochna Ginter-Kramarczyk

**Affiliations:** 1 Electrical Engineering Department, College of Engineering, Najran University Saudi Arabia, Najran 11001, Saudi Arabia; miditta@nu.edu.sa (M.I.); srrahman@nu.edu.sa (S.R.); omar2007_ahu@yahoo.com (O.A.); fsalkhtani@nu.edu.sa (F.S.A.); 2 Department of Physics, University of Agriculture, Faisalabad 38040, Pakistan; fareedazaheer580@gmail.com; 3 Department of Chemistry, University of Okara, Okara 56300, Pakistan; humaira0949@gmail.com; 4 Faculty of Mechanical Engineering, Poznan University of Technology, 60-965 Poznan, Poland; stanislaw.legutko@put.poznan.pl; 5 Department of Pharmaceutical Chemistry, College of Pharmacy, Najran University, Najran 11001, Saudi Arabia; matermaha@gmail.com; 6 Empty Qaurter Research Unit, Chemistry Department, College of Science and Art at Sharurah, Najran University Saudi Arabia, Najran 61441, Saudi Arabia; mabkhoot.alsaiari@gmail.com; 7 Civil Engineering Department, College of Engineering, Najran University Saudi Arabia, Najran 61441, Saudi Arabia; aaghanim@nu.edu.sa; 8 Mechanical Engineering Department, College of Engineering, Najran University Saudi Arabia, Najran 61441, Saudi Arabia; mkkhan@nu.edu.sa; 9 Faculty of Environmental Engineering and Energy, Department of Water Supply and Bioeconomy, Poznan University of Technology, 60-965 Poznan, Poland; izabela.kruszelnicka@put.poznan.pl (I.K.); dobrochna.ginter-kramarczyk@put.poznan.pl (D.G.-K.)

**Keywords:** manganese ferrite, amine functionalization, heavy metals, wastewater, adsorption isotherms

## Abstract

This study is focused on the kinetics and adsorption isotherms of amine-functionalized magnesium ferrite (MgFe_2_O_4_) for treating the heavy metals in wastewater. A sol-gel route was adopted to produce MgFe_2_O_4_ nanoparticles. The surfaces of the MgFe_2_O_4_ nanoparticles were functionalized using primary amine (ethanolamine). The surface morphology, phase formation, and functionality of the MgFe_2_O_4_ nano-adsorbents were studied using the SEM, UV-visible, FTIR, and TGA techniques. The characterized nanoparticles were tested on their ability to adsorb the Pb^2+^, Cu^2+^, and Zn^2+^ ions from the wastewater. The kinetic parameters and adsorption isotherms for the adsorption of the metal ions by the amine-functionalized MgFe_2_O_4_ were obtained using the pseudo-first-order, pseudo-second-order, Langmuir, and Freundlich models. The pseudo-second order and Langmuir models best described the adsorption kinetics and isotherms, implying strong chemisorption via the formation of coordinative bonds between the amine groups and metal ions. The Langmuir equation revealed the highest adsorption capacity of 0.7 mmol/g for the amine-functionalized MgFe_2_O_4_ nano-adsorbents. The adsorption capacity of the nanoadsorbent also changed with the calcination temperature. The MgFe_2_O_4_ sample, calcined at 500 °C, removed the most of the Pb^2+^ (73%), Cu^2+^ (59%), and Zn^2+^ (62%) ions from the water.

## 1. Introduction

Water contamination with heavy metals mainly occurs due to anthropogenic or natural processes. Since the production of metals from industrial activities is growing over time, the issue of waste management is worsening; dedicated efforts are needed to find economical solutions [1]. The heavy metals in wastewater are treated through chemical coagulation, chemical precipitation, photocatalytic degradation, flocculation, electrochemical routes, ion exchange, adsorption, membrane filtration, and bioremediation [2,3,4,5]. Most accessible technologies, on the other hand, may have technical and economic limitations, i.e., high capital and operating costs, sensitivity to operational conditions, large energy use, or sludge formation. Taking these considerations into account, adsorption is considered an economical and effective approach to eliminating heavy metal ions from water [6,7,8]. Surface atoms govern the adsorption of heavy metals by an adsorbent. The adsorbent surface becomes unstable and active as the number of surface atoms rises and, therefore, produces many unsaturated bonds [9].

Conventional adsorbents provide a limited number of active sites, a small surface area, and complex adsorption kinetics and post-adsorption separation mechanisms. The difficulty of separating the adsorbents from the treated solution limits their use in wastewater treatment technology [10]. On the other hand, magnetic nanoparticles become supermagnetic under 25 nm. These materials offer a large active surface area and can be separated from the water by subjection to an external magnetic field. However, magnetic adsorbents may undergo surface poisoning and a reduction in magnetic field strength due to the dissolution of the magnetic core in acidic environments and the agglomeration of magnetic adsorbent particles. This issue can be addressed by modifying the adsorbent surface and preventing the dissolution of the magnetic core in the solution. In many cases, modified surfaces negatively affect the activity of the adsorbent nanoparticles. To develop good synergy between the physiochemical characteristics of the adsorbent and the adsorption capacity, the modification approaches should be researched further to improve understanding the mechanism involved. This discussion suggests that surface modification is critical for increasing selectivity and removal efficiency [11].

The level of surface modification also depends on the pre-functionalization and calcination of the magnetic adsorbent nanoparticles. The effect of calcination temperature on the surface functionalization of magnetic adsorbent nanoparticles and MgFe_2_O_4_ nanoparticles, in particular, is not reported in the published literature. Herein, the role of the calcination temperature in the amine functionalization of MgFe_2_O_4_ nanoparticles in the context of the adsorption of heavy metals is assessed. An increase in calcination temperature may influence the particle size and exothermic oxidation of the adsorbent particles and, consequently, their level of functionalization. The density of the adsorbent nanoclusters may also change on heating due to oxidation, the recrystallization of the clusters, and the dissolution of impure products. The oxidation and crystal reformation govern the inter-grain spacing, surface activation, and surface porosity of the adsorbent particles. The sol-gel technique produces more remarkable results when developing ultrafine particles at reasonably low temperatures than the other available methods [12]. It is applied due to its unmatchable benefits, such as its short rate of reaction, energy efficiency, nano-sized powders, ease of operation, and limited aggregation of particles [13]. In the reported work, MgFe_2_O_4_ was produced using the sol-gel method and functionalized by utilizing ethanolamine as a surface modifier. This study aimed to investigate the characteristics of the absorption of heavy metals from wastewater by magnesium ferrite nanoparticles.

## 2. Materials and Methods

### 2.1. Synthesis of Ferrite Nanoparticles

The analytical-grade reagents used in this work were procured from Sigma-Aldrich. These reagents were used as supplied without any processing or additional treatment. First of all, 1.6 g of iron(III) nitrate nonahydrate and 0.5 g of magnesium nitrate hexahydrate [Mg(NO_3_)_2_.6H_2_O] were added to 40 mL of DI water. Next, 1.8 g of citric acid monohydrate [C_6_H_8_O_7_.H_2_O] was added to 40 mL of DI water and added into the initial aqueous solution. The solution was then stirred continuously at 60 °C. The solution pH was adjusted to 7 by dropwise addition of sodium hydroxide (NaOH) to the mixture. The mixture converted into sol on heating, which changed to gel after stirring for 3 h. The obtained gel was dried in an oven at 90 °C for 7 h. Subsequently, the dry gel was calcined in a furnace at 500 °C, 600 °C, and 700 °C, labeled as samples A, B, and C. After calcination, the samples were ground into a fine powder. The ferrite powder was characterized and tested for adsorption of heavy metals.

### 2.2. Characterization Techniques

FTIR, SEM, TGA, and UV-vis techniques were used to analyze the pristine and amine-functionalized adsorbents. An Agilent Cary-630 spectrometer was employed to produce FTIR spectra of the adsorbents in the range of 4000–500 cm^−1^. A scanning electron microscope (Nano-SEM 450) was used to produce SEM micrographs for evaluating the surface morphology of the adsorbents. A SEM microscope, featuring a STEM detector with a standard TEM grid, was used for transmission electron microscopy analysis. STEM analysis was used to determine particle size and morphology in support of SEM analysis. A TGA/DSC instrument was operated to get thermogravimetry profiles of pristine and functionalized adsorbents for the analysis of the amount of amine coating during the functionalization of the adsorbent.

### 2.3. Amine Functionalization of Ferrite Nanoparticles

The ethanolamine was used as a surface modifier for the functionalization of MgFe_2_O_4_ nanoparticles. About 1.5 g of MgFe_2_O_4_ and 2 mL ethanolamine were added to 20 mL of distilled water. After 30 min of stirring and heating, the aqueous liquid was cooled to room temperature. To wash the precipitates and maintain a pH of 7, deionized water was used. The washed sample was dried in an oven for 2 h. All samples were amine-functionalized using the same procedure. The functionalization procedure was completed by following the catalyst-free method reported by Gu et al. [14] and Nonkumwong et al. [15] in different studies.

### 2.4. Adsorption of Heavy Metals

Precisely 0.02 g of lead nitrate, 0.02 g of copper chloride dehydrate, and 0.02 g of zinc acetate dehydrate were added to deionized water (50 mL) to simulate wastewater. The adsorption experiments were conducted by dissolving 0.25 g of MgFe_2_O_4_-NH_2_ adsorbent in the prepared wastewater. The pH of the aqueous mixture was maintained at 7. This aqueous mixture was stirred for 90 min and supernatants were filtered. The residual metal content in supernatants was measured using atomic absorption spectroscopy of the samples. The agitation period and initial concentrations of the heavy metals might be varied to have a good understanding of the adsorption response of the magnetic nano-adsorbent. The kinetic parameters and adsorption isotherms for the selected heavy metals onto the amine-functionalized MgFe_2_O_4_ were calculated using different chemical models [16].

## 3. Results and Discussion

### 3.1. Metal Ion Adsorption Mechanism

**Scheme 1:**
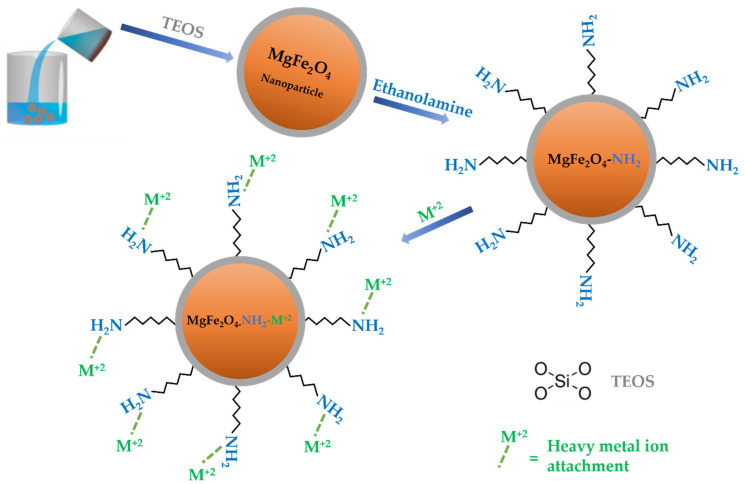
Illustration of amine-functionalization and metal ions adsorption onto MgFe_2_O_4_-NH_2_ adsorbent.

Tetraethyl orthosilicate (TEOS) silane was used for the direct silanization of the surface of the magnetic adsorbent. On the surface, the silica produces a highly robust covalent coating with grafted functional groups. It enables the additional functionalization of the core functional groups, resulting in a wide range of surface functionalities for adsorption applications [17]. The silica coating also prevents the agglomeration of the adsorbent particles. The silica-coated adsorbent was amino-functionalized using ethanolamine. A reaction scheme involving the amine functionalization of the MgFe_2_O_4_ adsorbent for the adsorption of the heavy metal ions is presented as Scheme 1. The mechanism of metal ions’ adsorption onto the adsorbent heavily depends on functional groups, which give binding affinity to the metal ions. The deposition of pollutants on the MgFe_2_O_4_-NH_2_ adsorbent in a multicomponent solution may occur through a physical process or the formation of chemical bonds. The −NH_2_ groups on the functionalized MgFe_2_O_4_ nanoparticles can be protonated and deprotonated, depending on the acidic and basic environment.

Since −NH_2_ groups possess a hard basic nature, they can bind strongly with heavy metal cations. As shown by the processes below, the ions in the aqueous solution can be in the form of hydrolysis or solvation species:

$$M^{2+}+{\mathrm{nH}}_{2}\overset{}{↔}M{\left(H_{2}O\right)}_{n}^{2+}\;M{\left(H_{2}O\right)}_{n}^{2+}\;\overset{}{↔}M{\left(H_{2}O\right)}_{}^{n-1}+H^{+}\;{\mathrm{nM}}^{2+}+{\mathrm{mH}}_{2}O\;\overset{}{↔}M_{n}{\left(\mathrm{OH}\right)}_{m}^{2n-m}+{\mathrm{mH}}^{+}$$

The mass transfer may occur due to pore filling, ion exchange, hydrophobic interaction, and H-bonding. The adsorption by the MgFe_2_O_4_-NH_2_ adsorbent increases as the number of surface atoms increases, making them more active and unstable, as well as providing more unsaturated bonds. The M^2+^ ions dominate when the solution pH is below 5, while M(OH)^+^ tends to initiate when the solution pH is greater than 5. The M(OH)_2_ is present when the solution pH exceeds 6.5. Since the solution pH was maintained at 7 in this study, the formation of the M(OH)_2_ was anticipated. The rapid adsorption of the M^2+^ ions onto MgFe_2_O_4_-NH_2_ reveals that chemical adsorption dominates the physical adsorption, most likely due to the complexation of the -NH_2_ groups and M^2+^ ions. The pseudo-second order described the adsorption of the heavy metal ions onto the adsorbent surface due to the formation of chemical bonding between the polar functional groups and the metal ions. The chemical bonding resulted in an adsorbent with a high capacity for cation exchange. The findings suggested that the adsorption of heavy metal ions onto MgFe_2_O_4_-NH_2_ adsorbent is driven by the formation of coordinate bonds or chemisorption.

### 3.2. FTIR and TGA Analysis of Amine-Functionalized Adsorbent

**Figure 1:**
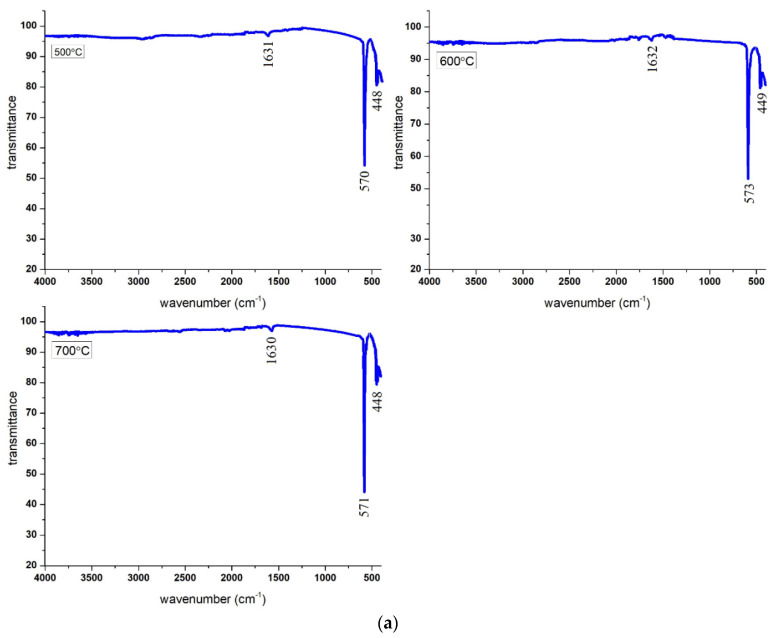

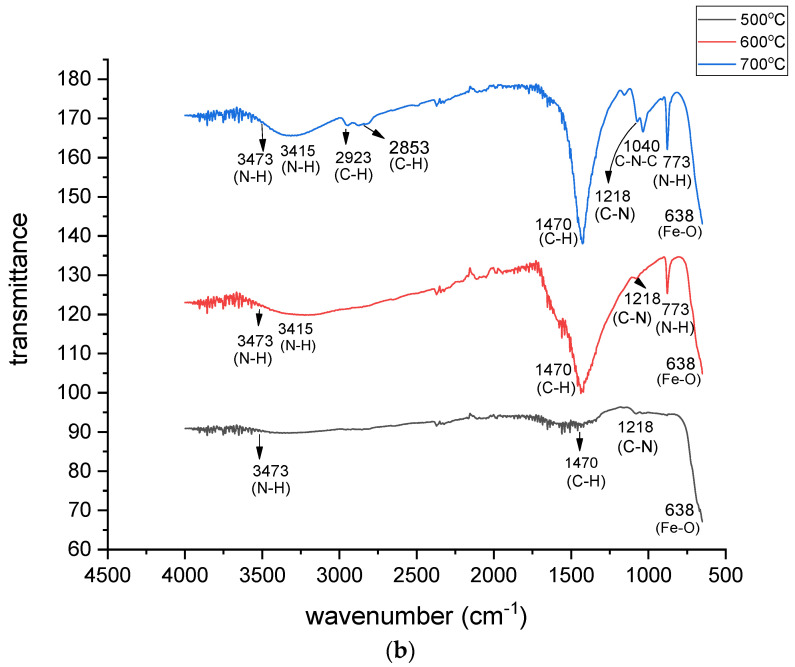
(**a**) FTIR spectra of pristine MgFe_2_O_4_ adsorbent calcined at different temperatures. (**b**) FTIR spectra of amine-functionalized magnesium ferrite nanoparticles calcined at different temperatures.

The attachment of the amine groups onto the MgFe_2_O_4_ adsorbent was characterized by the production of the FTIR spectra of the pristine and amine-treated adsorbents. Figure 1a reports the FTIR profiles of the pristine adsorbents calcined at 500 °C, 600 °C, and 700 °C. The FTIR spectra did not vary significantly with the change in calcination temperature. Three curves at 500 °C, 600 °C, and 700 °C show the same number of peaks. However, the intensity of the characteristic absorption peaks of the M–O band slightly increased with the calcination temperature. The formation of the spinel structure of the MgFe_2_O_4_ adsorbent with cation and anion distribution on the octahedral and tetrahedral lattice sites was confirmed by the FTIR analysis. Two absorption bands were noticed in the spinel structure of the MgFe_2_O_4_ adsorbent, at 570 cm^−1^ and 450 cm^−1^. The high-frequency band revealed the M–O bond stretching vibration at the tetrahedral sites (A), while the low-frequency band revealed metal-oxygen stretching vibration at the octahedral sites (B). These vibrations were attributed to the shorter bond length of the metal–oxygen link in the tetrahedral sites and the larger metal–oxygen bond length in the octahedral sites. A bending vibration mode of O–H was seen at 1630 cm^−1^, indicating both free and absorbed water [18]. The length of the metal oxide bonds in each site caused a shift in the placement of the tetrahedral and octahedral bands. The presence of Fe^2+^ cations on the octahedral sites was confirmed by a shoulder near the octahedral absorption band. The cations caused the absorption band to split due to the Jahn–Teller phenomenon, which occurred due to local deformations in the lattice [19].

The MgFe_2_O_4_ samples were amine-functionalized after calcination at different temperatures. No calcination was performed after the amine functionalization of the adsorbent samples. Therefore, the FTIR spectra of the functionalized adsorbents and the level of functionalization are discussed based on the calcination of the pristine samples. Figure 1b shows that there was a small difference between the FTIR spectra of the MgFe_2_O_4_-NH_2_ samples obtained after the calcination of the pristine MgFe_2_O_4_ at 600 °C and at 700 °C. However, the FTIR spectrum of the MgFe_2_O_4_-NH_2_ nanoparticles at 500 °C was different from the other two spectra. The FTIR spectra of the MgFe_2_O_4_-NH_2_ nanoparticles and the pristine calcined at 500 °C, differed because at low temperatures, the active sites on the surfaces of the particles were low in number and no definite sharp peaks were formed. As the temperature increased, more active sites were produced on the nanoadsorbent surface, revealing the attachment of the functional groups. A rise in calcination temperature might have impacted the particle size and exothermic reactions of the oxidation of the adsorbent particles and, consequently, the level of functionalization. The density of the adsorbent nanoclusters increased on heating due to the occurrence of oxidation, the recrystallization of the clusters, and the removal of the impure products at high temperatures. The oxidation and crystal reformation enhanced the inter-grain spacing and surface porosity of the adsorbent particles. Therefore, as the calcination temperature of the pristine adsorbent increased, extra active sites were produced on the adsorbent surface, revealing the attachment of an excessive number of functional groups.

The creation of the Fe-O stretching mode by the MgFe_2_O_4_ is marked by a spectral line at 638 cm^−1^, which confirms the spinel structure of the adsorbent. An FTIR peak at 773 cm^−1^ indicates the N-H wagging mode. The Fe-O vibrations masked the Fe–O–Si bonds in the FTIR spectrum. Similarly, an FTIR peak at 1040 cm^−1^ shows the formation of the C-N-C asymmetric stretching mode. This mode also shows C−O overlapping with C−N stretching vibration, which offers confirmation of the attachment of the amine-functional groups on the adsorbent. The peak around 1218 cm^−1^ reflects the C-N stretching mode. The peak around 1470 cm^−1^ indicates the C-H scissoring mode. The pair of peaks at 3415 and 3473 cm^−1^ represents the N-H stretching of athe mine, which confirms the grafting of the amine onto the MgFe_2_O_4_ nanoparticles. These peaks are missing in the FTIR spectra of the pristine MgFe_2_O_4_ nanoparticles, as shown in Figure 1a. The peaks at 2923 cm^−1^ and 2853 cm^−1^ show the C−H stretching vibration bond in the ethanolamine. The low-frequency peaks in the 500−850 cm^−1^ range show the stretching modes of the Fe−O and Mg−O in the spinel MgFe_2_O_4_.

**Figure 2:**
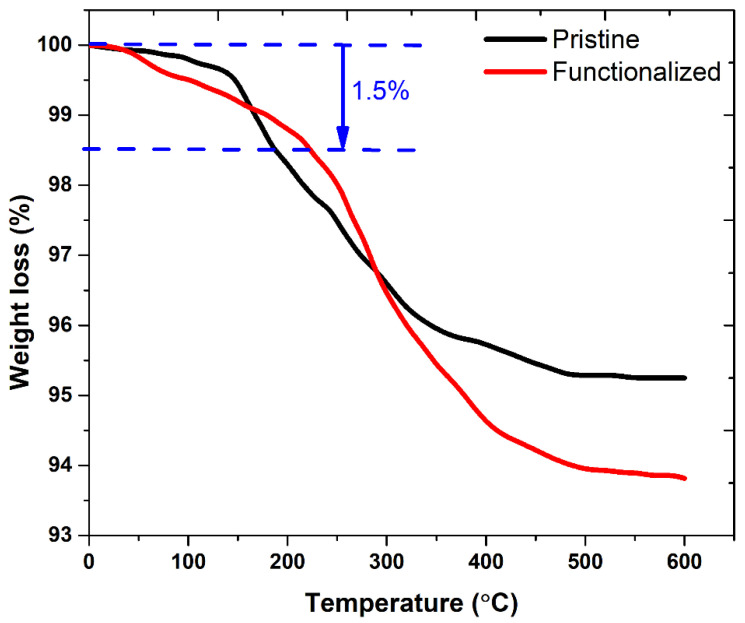
TGA profiles of pristine and amine-functionalized adsorbents.

The TGA plots in Figure 2 were analyzed to calculate the amount of amine on the surface of the functionalized adsorbent. The sample was heat-treated by raising the temperature from ambient to 600 °C at a fixed rate of 10 °C/min. To quantify the decomposition of the amine coating on the adsorbent surface, the weight loss of the pristine adsorbent was compared to that of the functionalized adsorbent during heating. The weight loss began at 100 °C and continued until 600 °C. The weight loss was rapid between 200 °C and 400 °C, before gradually decreasing to a steady state. The pristine and amine-functionalized adsorbents exhibited weight losses of 4.7% and 6.2%, respectively. The weight loss of the pristine adsorbent was mainly due to evaporation of moisture content in the adsorbent through chemical and physical processes and the stabilizer used to stabilize the particle size. The weight loss of the functionalized adsorbent was higher than that of the pristine adsorbent due to the decomposition of the functional groups conjugated on the adsorbent surface [20]. The amount of amine groups attached to the adsorbent was measured at about 1.5%. The amount of amine groups was measured from the difference in the initial weight losses (%) of the pristine and functionalized adsorbent nanoparticles at a temperature below 300 °C. The difference in weight loss values was equal to the amount of functional groups and bound water. Thus, the FTIR and TGA analyses confirmed that the magnetic adsorbent was functionalized with amine groups. The functionalized adsorbent was highly stable in water due to the H-bonding between the water and the amine groups of the functionalized adsorbent [21].

### 3.3. UV-Visible Analysis

**Figure 3:**
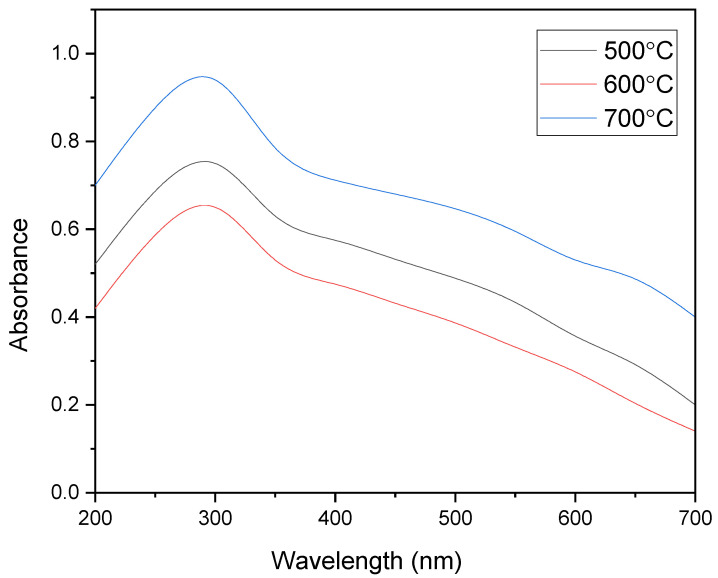
UV absorption profiles of pristine MgFe_2_O_4_ nanoparticles calcined at different temperatures.

**Figure 4:**
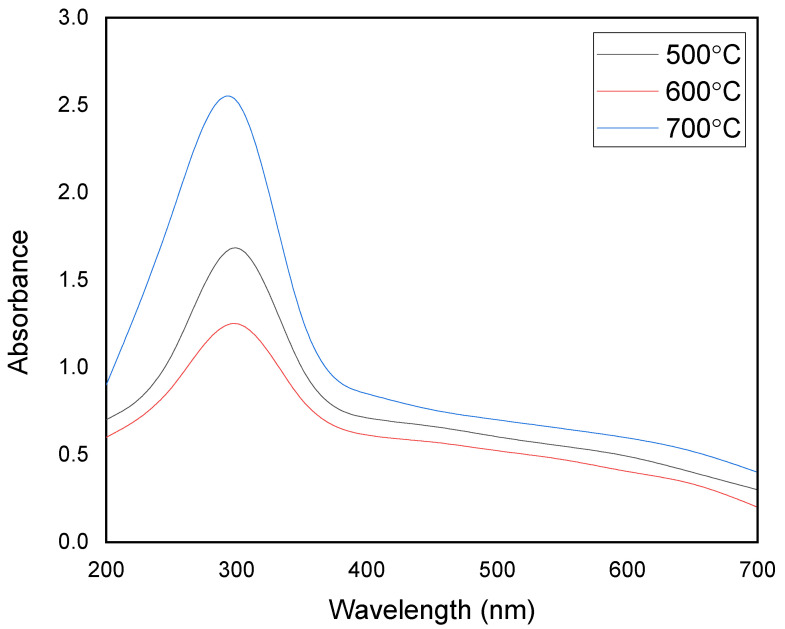
UV absorption profiles of MgFe_2_O_4_-NH_2_ samples calcined at different temperatures.

UV-visible spectroscopy was conducted to study the optical properties of the synthesized sample. The UV-visible absorption spectra of the MgFe_2_O_4_ and MgFe_2_O_4_-NH_2_ nanoparticles, calcined at different temperatures, are reported in Figure 3 and Figure 4, respectively. These spectra reveal high absorption in the ultraviolet and visible regions. The bandgap energy was determined from the expression of the absorption coefficient near the band edge by supposing a direct bandgap using Tauc relation, given as follows:

$$E_{g}=\frac{h.c}{\lambda_{max}}$$

where, *h* is the Plank constant, *λ_max_* is the wavelength at the absorption edge, and *c* is the speed of light. The *E_g_* values of the pristine MgFe_2_O_4_ nanoparticles, calcined at 500 °C, 600 °C, and 700 °C, were calculated at around 2.05 eV, 2.03 eV, and 2.00 eV, respectively. The *E_g_* values of the MgFe_2_O_4_-NH_2_ nanoparticles were calculated at around 2.09 eV, 2.08 eV, and 2.06 eV, respectively. The bandgap energy decreased as the temperature of the calcination increased in both the pristine and amine-functionalized adsorbents. The crystallite growth of the synthesized products was most probably due to the lowering of the bandgap energy at high calcination temperatures. The UV spectra were measured by preparing the suspensions of the pristine and functionalized nanoparticles. The bandgap energy and UV absorption of the adsorbent nanoparticles were found nearly in the visible region. This showed that the light absorption partly dominated the light-scattering part, since scattering is inversely proportional to the wavelength. The longer the wavelength, the lower the scattering factor.

The MgFe_2_O_4_-NH^2^ samples showed a stronger absorbance peak than the pristine MgFe_2_O_4_ nanoparticles. The ethanolamine-functionalized MgFe_2_O_4_ nanoparticles were expected to enhance the absorbance peak intensity due to the deposition of the nonmagnetic amino groups on the adsorbent. It was revealed that the absorbance intensity depends on the number of reactive −NH_2_ groups attached to the surface. The amount of −NH_2_ group on the nanoparticle surface increases with the calcination temperature due to increased surface roughness and the porosity of the nanoparticles.

## 4. Morphology and Size Distribution

**Figure 5:**
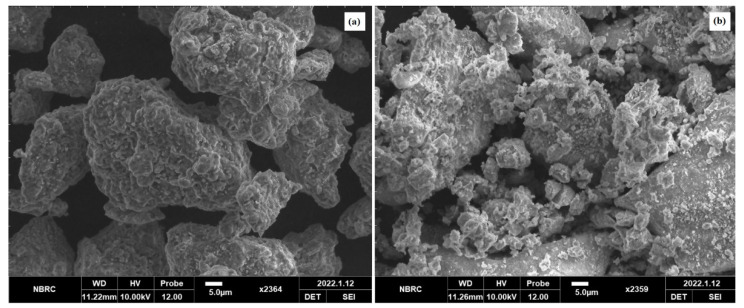

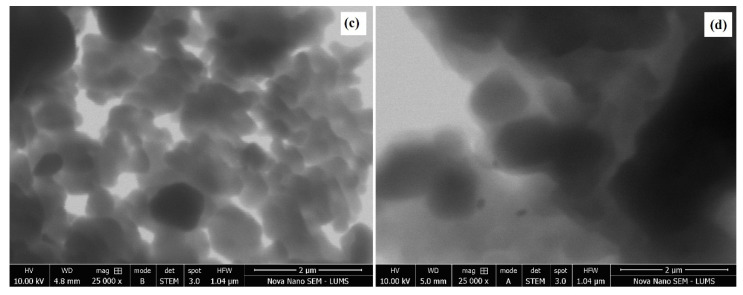
(**a**) SEM micrograph of pristine MgFe_2_O_4_, (**b**) SEM micrograph of amine-functionalized MgFe_2_O_4_, (**c**) STEM micrograph of pristine MgFe_2_O_4_, (**d**) STEM micrograph of amine-functionalized MgFe_2_O_4_.

**Figure 6:**
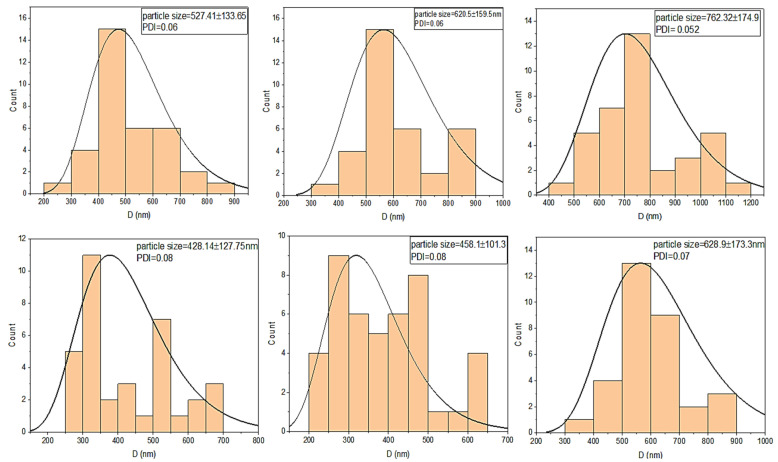
Particle-size histograms of pristine and amine-functionalized MgFe_2_O_4_ nanoparticles calcined at different temperatures.

SEM images were produced to elaborate the morphology and size of the spinel MgFe_2_O_4_ nanoparticles. Figure 5a,b compares the SEM morphologies of the pristine and amine-functionalized MgFe_2_O_4_ nanoparticles at 700 °C. The pristine MgFe_2_O_4_ nanoparticles were uniformly dispersed, less agglomerated, and homogeneous, with an average particle size slightly larger than that of the functionalized nanoparticles. The silanization and functionalization processes prevented the agglomeration of the nanoparticles. The calcination temperature did not cause significant changes in surface morphology. As shown in Figure 6, the particle size of the pristine MgFe_2_O_4_ at 500 °C, 600 °C, and 700 °C was measured at about 527.41 nm, 620.5 nm, and 762.32 nm, respectively. The particle size slightly decreased after the amine functionalization. The pre- and post-functionalization morphologies of MgFe_2_O_4_ were assessed further by producing STEM images, as shown in Figure 5c,d. The STEM images showed a slight change in morphology after functionalization. The shapes and boundaries of the nanoparticles were clearer compared to the pristine nanoparticles. A thin coating of functional material on the nanoparticle surface can also be seen in the form of light shade circling the nanoparticles.

These results show that the functionalization of MgFe_2_O_4_ nanoparticles can reduce the agglomeration of pristine ferrite nanoparticles. The grain sizes of the amine-functionalized MgFe_2_O_4_ at different temperatures were measured at about 428.14 nm, 458.1 nm, and 628.9 nm, respectively. Some of the histograms, presented in Figure 6, showed a slightly bimodal distribution in the particle sizes, as depicted by major and minor modes. The bimodal distribution was not important in our case, since we determined the average size of one class of particles. The bimodal distribution is only important when the sizes of different species or types of particles are being studied. This distribution is commonly used to examine individual size subpopulations to better understand individual distributions.

### 4.1. Adsorption Kinetics

The pseudo models were developed to understand the adsorption kinetics. In reversible reactions in which an equilibrium is maintained between the solid and liquid phases, the pseudo-first-order kinetics are utilized, implying physisorption instead of chemisorption. The kinetic process of heavy metal ions’ adsorption onto the adsorbents is explained through the pseudo-second-order model. The cation exchange capacity of adsorbents is influenced by a chemical interaction among the surface functional groups and heavy metal ions.

For the study of kinetic parameters, the adsorbent is added to heavy water and the resulting solution is stirred. The agitation time is changed and the corresponding initial and final metal ion concentration in the aqueous solution is monitored. At a certain point in time, the concentration does not change further. At this point, the final concentration is equal to the equilibrium concentration. The adsorption capacity (*q_e_*) is determined by defining *C_f_* as *C_e_* in the following formula:

$$q_{t}=\frac{\left(C_{i}-C_{f}\right)V}{m}$$

where $q_{t}$ is the quantity of metal ions adsorbed by the adsorbent, $C_{i}$ is the initial concentration of heavy metals in the aqueous solution, and $C_{f}$ is the final concentration of heavy metals. The volume of heavy metals in the solution is represented by V, while *m* represents the mass of the dry adsorbent in grams. For the pseudo-first-order model, the graph is plotted between t and log(*q_e_* − *q_t_*), and the resulting line equation is compared with the integrated linear form of the pseudo-first-order model. The slope and intercept of the line is used to calculate the pseudo-first-order parameters. For the pseudo-second-order model, the graph is plotted between *t* and *t*/*q_t_* and the resulting line equation is compared with the integrated linear form of the pseudo-second-order model. The slope and intercept of the line is used to calculate the pseudo-first-order parameters.

For plotting isotherm models, the parameters *C_e_* and *q_e_* are used. *C_e_* is the concentration of the adsorbate in the aqueous solution after adsorption. From *C_e_*, *q_e_* is calculated by using the above equation. For Langmuir isotherms, the graph is plotted between *C_e_* and *C_e_*/*q_e_*, and the resulting line equation is compared with the linear form of the Langmuir isotherm model. The slope and intercept of the line is used to calculate the isotherm parameters. For Freundlich model, the graph is plotted between log *C_e_* and logq_e_, and the resulting line equation is compared with the linear form of the Freundlich model. The slope and intercept of the line is used to calculate the Freundlich parameters. In the present work, the pseudo-second-order model fit the best. This model suggests chemisorption, i.e., chemical changes occur when the adsorbate binds with the adsorbent. As a result, heavy metal ion adsorption onto the prepared sample occurred via chemisorption rather than physisorption.

### 4.2. Pseudo-First-Order Kinetics

The pseudo-first-order model is premised on the idea that a change in the solute take-up rate can have a direct relationship with the difference in the saturation concentrations and the amount of solid take-up with time, and it is widely applicable during the first phase of adsorption processes. For adsorption, occuring via diffusion through an interface, the kinetics are typically observed to fit pseudo-first-order rate equation. The linear form of the pseudo-first-order model is as follows.

$$\frac{dq_{t}}{dt}=k_{1}\;(q_{e}-q_{t})$$

where *q_t_* and *q_e_* (milligram/gram) represent the quantity adsorbed at “*t*” time and at equilibrium, respectively, while *k_1_* denotes the pseudo-first-order model’s constant at equilibrium. After applying the integration and boundary conditions, the above equation becomes:

$$log(q_{e}-q_{t})=log(qe)-\frac{k_{1}}{2.303}\;t$$

The linear graphs of the *log(q_e_ − q_t_)* over time are used to find the k_1_ value. If adsorption obeys first-order kinetics, the intercept of the *log(q_e_ − q_t_)* against the *t* plot is equal to *log(q_e_).* For slower adsorption, the correct equilibrium condition can be difficult to attain, making a precise determination of *q_e_* extremely difficult [22].

### 4.3. Pseudo-Second-Order Kinetics

The pseudo-second-order kientics are premised on the idea that chemisorption is a rate-limiting phase, which predicts adsorption behavior throughout the adsorption process. The adsorption capacity, rather than the adsorbate concentration, determines the rate of adsorption in this situation. The main benefit of this model over the pseudo-first-order model is that the adsorption capacity at equilibrium may be derived from this model, eliminating the requirement of measuring the equilibrium adsorption capacity via experiment. For pseudo-second-order kinetics, the differential expression is as follows:

$$\frac{dq_{t}}{dt}=k_{2}\;{\left(q_{e}-q_{t}\right)}^{2}$$

After the mathematical elaboration and consideration of the boundary condition, the pseudo-second-order model is:

$$\frac{t}{q_{t}}=\frac{1}{k_{2}q_{e}^{2}}+\frac{t}{q_{e}}$$

with

$$h=k_{2}q_{e}^{2}\;$$

where h denotes the initial sorption rate, *q_e_* (milligram/gram) represents the equilibrium quantity of the adsorbed dye, and *k*_2_ represents the pseudo-second-order model rate-constant (gram/milligram.minute). A direct relationship should emerge when plotting t/q_t_ against t. We can determine the quantity of theadsorbed adsorbate at the equilibrium using the slope (1/*q_e_*) and intercept (1/h or 1/*k*_2_*q_e_*^2^), which yields the second-order rate constant, *k*_2_ [22].

### 4.4. Pseudo Models for Adsorption of Cu^2+^ onto MgFe_2_O_4_-NH_2_ Nanoparticles

**Figure 7:**
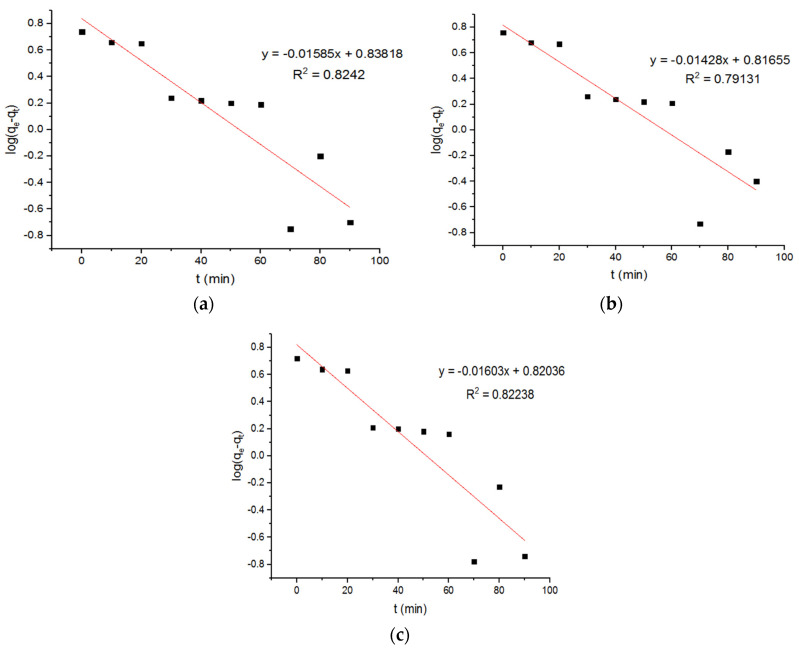
Pseudo-first-order adsorption plots of Cu^+2^ ions onto MgFe_2_O_4_-NH_2_ nanoparticle pre-functionalization-calcined at (**a**) 500 °C, (**b**) 600 °C, and (**c**) 700 °C.

**Figure 8:**
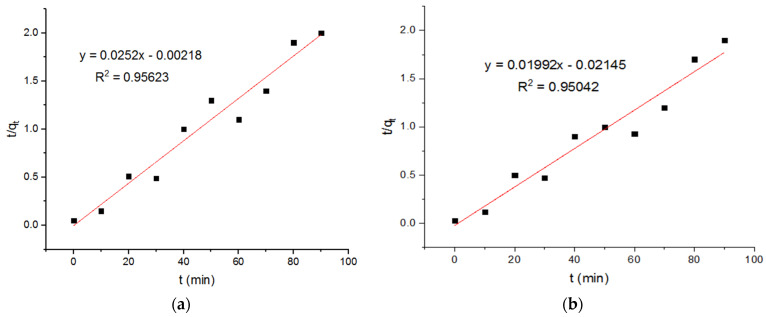

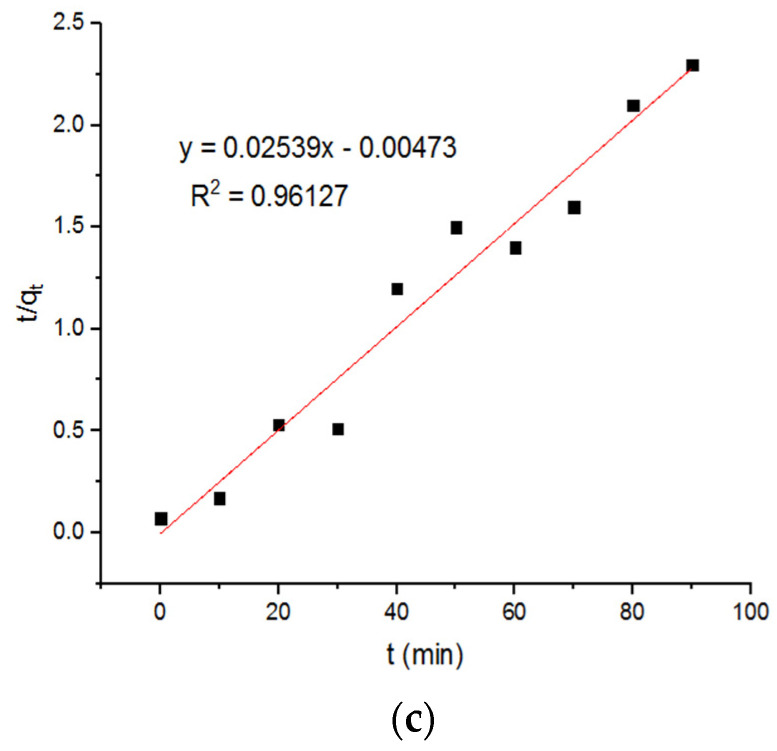
Pseudo-second-order adsorption plots of Cu^+2^ ions onto MgFe_2_O_4_-NH_2_ nanoparticles pre-functionalization-calcined at (**a**) 500 °C, (**b**) 600 °C, and (**c**) 700 °C.

It is reported that the amount of copper adsorbed on magnetic ferrite nanoparticles at equilibrium is about 2.32 mg/g. The pseudo-first-order kinetics were used in these calculations [23]. The value of *q_e_*, obtained using pseudo-second-order kinetics, is around 149.25 mg/g. Based on the pseudo-first-order model, the plots of the adsorption of the Cu^+2^ onto the MgFe_2_O_4_-NH_2_ nanoparticles are reported in Figure 7, and the subsequent computed kinetic parameters are reported in Table 1. It is shown that the kinetic parameters of the adsorbent, calcined at different temperatures, did not differ notably. For the second-order model, the plots of the Cu^2+^ adsorption onto the MgFe_2_O_4_-NH_2_ nanoparticles are shown in Figure 8, and the subsequent computed kinetic parameters are reported in Table 1. The pseudo-second-order relation yielded a better-fitting straight line when the correlation coefficient (R^2^) was taken into account. These results indicated that Cu^2+^ adsorption on amine-treated ferrite nanoparticles occurs through the chemisorption process.

### 4.5. Pseudo Models for Adsorption of Pb^2+^ onto MgFe_2_O_4_-NH_2_ Nanoparticles

**Figure 9:**
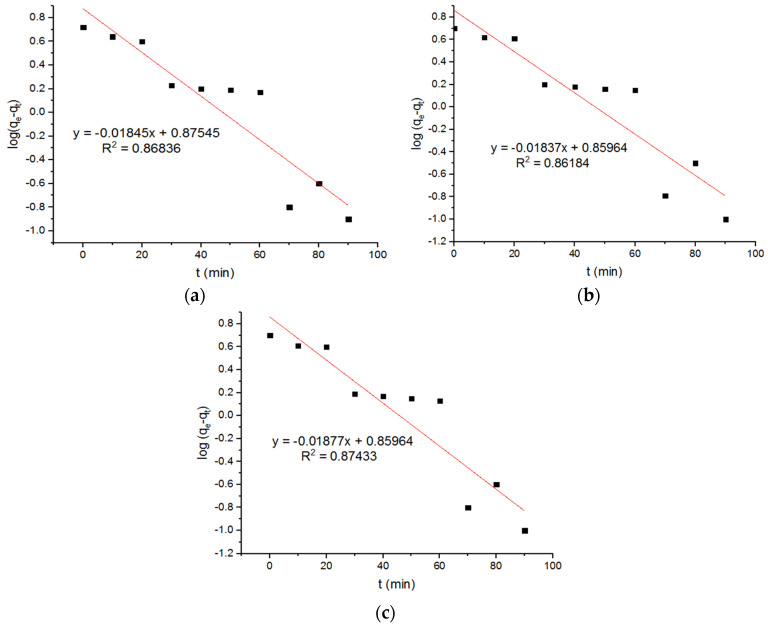
Pseudo-first-order plots for Pb^2+^ adsorption onto MgFe_2_O_4_-NH_2_ nanoparticles pre-functionalization-calcined at (**a**) 500 °C, (**b**) 600 °C, and (**c**) 700 °C.

**Figure 10:**
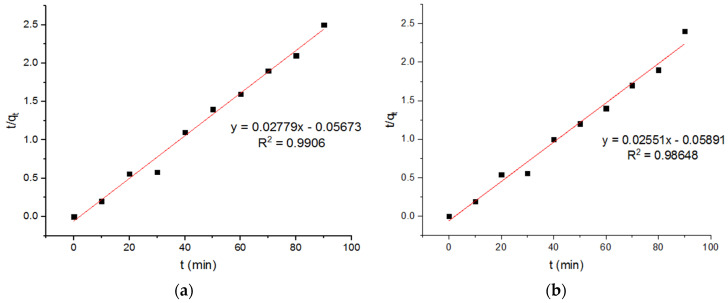

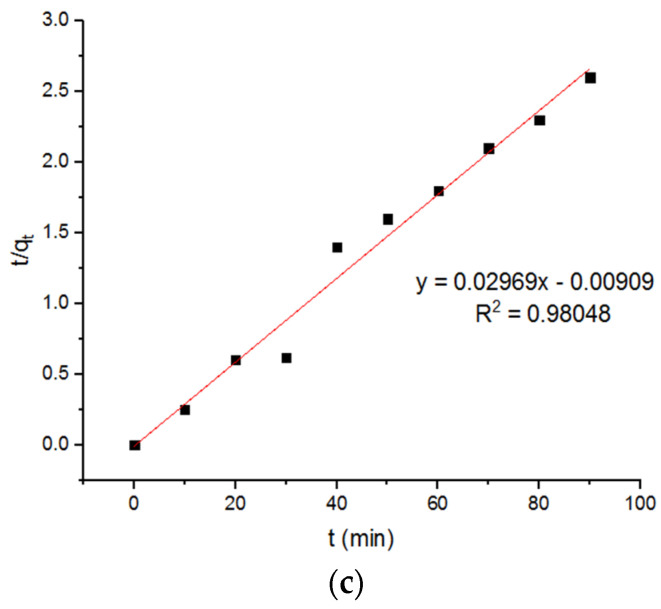
Pseudo-second-order plots of adsorption of Pb^2+^ onto MgFe_2_O_4_-NH_2_ nanoparticles pre-functionalization-calcined at (**a**) 500 °C (**b**) 600 °C, and (**c**) 700 °C.

Tran et al. [24] reported that the amount of lead adsorbed on Cu_0.5_Mg_0.5_Fe_2_O_4_ at equilibrium is about 3.66 mg/g. They used pseudo-first-order kinetics and calculated q_e_ at around 38.2 mg/g. The plots of adsorption the Pb^2+^ ions onto the MgFe_2_O_4_-NH_2_ nanoparticles, based on pseudo-first-order kinetics at pre-functionalization calcination temperatures, are reported in Figure 9, and the subsequent computed kinetic parameters are reported in Table 2. It is shown that the kinetic parameters at 500 °C and 700 °C did not differ significantly. Three pseudo-second-order plots for the adsorption of Pb^2+^ ions onto MgFe_2_O_4_-NH_2_ nanoparticles are reported in Figure 10, and the subsequent computed kinetic parameters are reported in Table 2. It is shown that the kinetic parameters at different calcination temperatures did not differ significantly.

### 4.6. Pseudo Models for Adsorption of Zn^2+^ onto MgFe_2_O_4_-NH_2_ Nanoparticles

**Figure 11:**
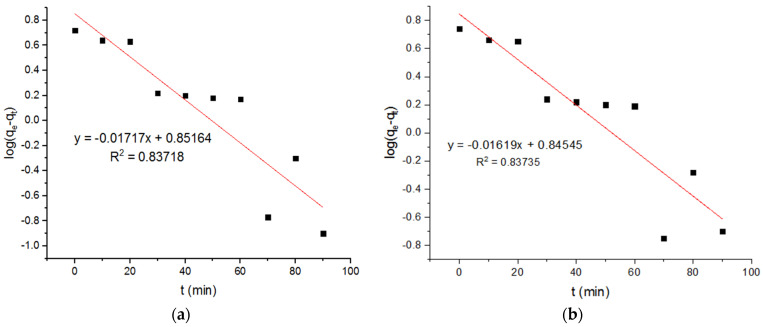

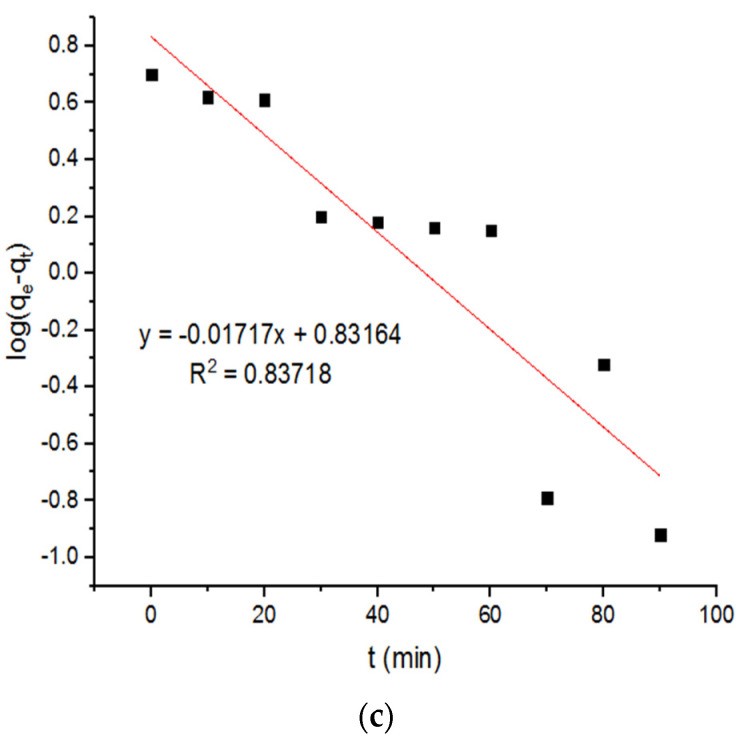
Pseudo-first-order plots for adsorption of Zn^2+^ ions onto MgFe_2_O_4_-NH_2_ nanoparticles pre-functionalization-calcined at (**a**) 500 °C, (**b**) 600 °C, and (**c**) 700 °C.

**Figure 12:**
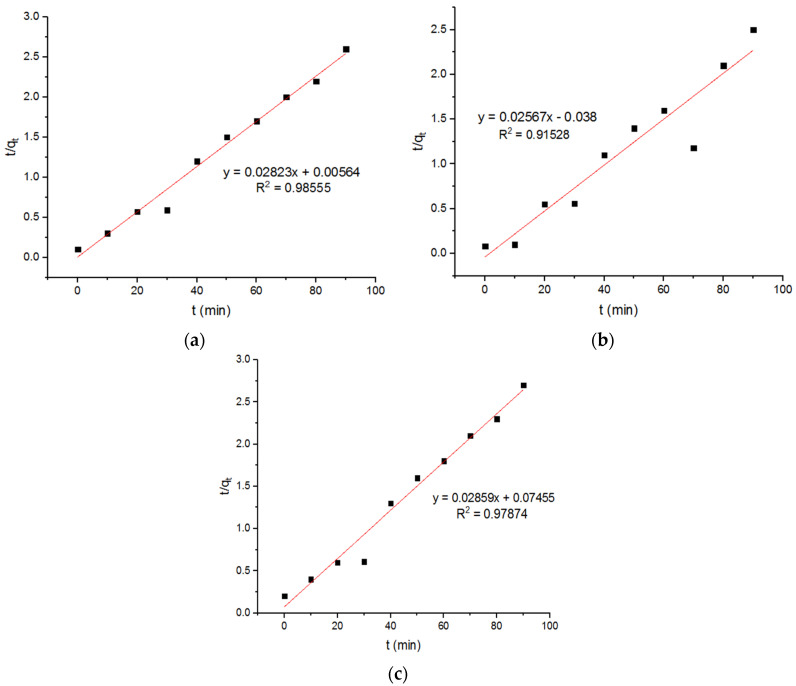
Pseudo-second-order plots for adsorption of Zn^2+^ ions onto MgFe_2_O_4_-NH_2_ nanoparticles pre-functionalization-calcined at (**a**) 500 °C, (**b**) 600 °C, and (**c**) 700 °C.

The plots of the pseudo-first-order and second-order adsorption of the Zn^2+^ ions onto the MgFe_2_O_4_-NH_2_ nanoparticles, pre-functionalization-calcined at different temperatures, are presented in Figure 11 and Figure 12, respectively. The subsequent kinetic parameters are reported in Table 3. It is shown that the kinetic parameters at different calcination temperatures did not differ significantly.

## 5. Adsorption Isotherms

Th adsorption isotherms were investigated utilizing both Freundlich and Langmuir isotherms. It was demonstrated how the adsorbent interacted with the adsorbates. The Langmuir model involves the homogeneous monolayered adsorption of pollutants onto the adsorbent by excluding the multilayer capillary-condensation. It simply indicates that the more binding sites are present on the surface of adsorbent, the more effective the adsorption. The Freundlich isotherm is well described for the surface of heterogeneous adsorbents, with many types of adsorption site.

### 5.1. Langmuir Model

The adsorbate and adsorbent remain in dynamic equilibrium in monolayer adsorptions and the Langmuir model. The Langmuir adsorption isotherm was created to explain gas-to-solid-phase adsorption, but it was later used to examine the solid–liquid interface. Fractional coverage is a measure of how much of the surface is covered, and it is determined by the adsorbate concentration. The mathematical evolution is based on a physical simplification of the mechanism regarding certain assumptions: (1) the surfaces are homogeneous, implying that almost all the sites are equivalent energetically, (2) adsorption is the monolayer procedure in which every site may only adsorb one adsorbate molecule, (3) adsorbed molecules do not network with each other laterally, and (4) adsorption can be reversed.

The Langmuir adsorption isotherm is expressed as:

$$q_{e}=\frac{K_{L}Q_{m}C_{e}}{1+K_{L}C_{e}}$$

where *C_e_* (mole/litter) and *Q_m_* (milligram/gram or mole/gram) indicate the equilibrium concentration and maximum adsorption capacity, respectively, and the Langmuir constant ‘*K_L_*’ is the adsorption energy or adsorbate–adsorbent constant at equilibrium. This equation can be transformed into linear form as:

$$\frac{1}{q_{e}}=\frac{1}{K_{L}Q_{m}C_{e}}+\frac{1}{Q_{m}}\;\frac{C_{e}}{q_{e}}=\frac{1}{K_{L}Q_{m}}+\frac{C_{e}}{Q_{m}}$$

Langmuir constants can be determined by plotting 1/*q_e_* against 1/*C_e_*, or the *C_e_*/*q_e_* against *C_e_* [22].

### 5.2. Freundlich Model

Reversible, non-ideal, multilayer adsorption at heterogeneous surfaces can be described by the Freundlich adsorption isotherm. The binding energies of adsorption sites differs according to the isotherm. Instead of uniform energy, the energy distributions at the adsorbing sites have spectra with varied binding energy values and obey the exponential function that is closer to the actual situation [25].

$$q_{e}=K_{F}C_{e}^{1/n}$$

where *C_e_* (mole/litter) is the adsorbate concentration under equilibrium. The dsorption intensity and the capacity of the adsorption are represented by the Freundlich constants 1/*n* and *K_F_*, respectively. By applying logarithms, the above equation can indeed be represented as:

*log*(*qe*) = *logK*_*F*_ + (1/*n*) *logC*_*e*_

where the *n* and *K_F_* parameters can be accessed by representing *log*(*q_e_*) as the function of *C_e_*. The 1/*n* value provides useful information about the adsorption processes. A 1/*n* value of less than one indicates chemisorption or a typical Langmuir adsorption isotherm, and a 1/*n* value greater than one indicates cooperative adsorption [22].

### 5.3. Isotherm Models for Cu^2+^ Adsorption onto MgFe_2_O_4_-NH_2_ Nanoparticles

Ivanets et al. [7] reported that the magnesium ferrite nano-adsorbents possessed a maximum capacity for Cu^2+^, calculated from the Langmuir equation, around 0.49 mmol/g. Liu et al. [23] found that the maximum adsorption capacity (q_m_) of magnetic ferrite nanoparticles for Cu^2+^ is about 124.8 mg/g. The value of R^2^ for the Langmuir isotherm is greater than the R^2^ value for the Freundlich adsorption isotherm. This indicates that the Langmuir isotherm is best fitted to the adsorption of copper (II) ions.

**Figure 13:**
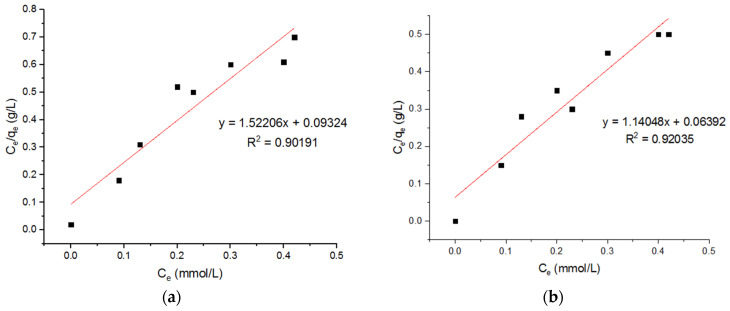

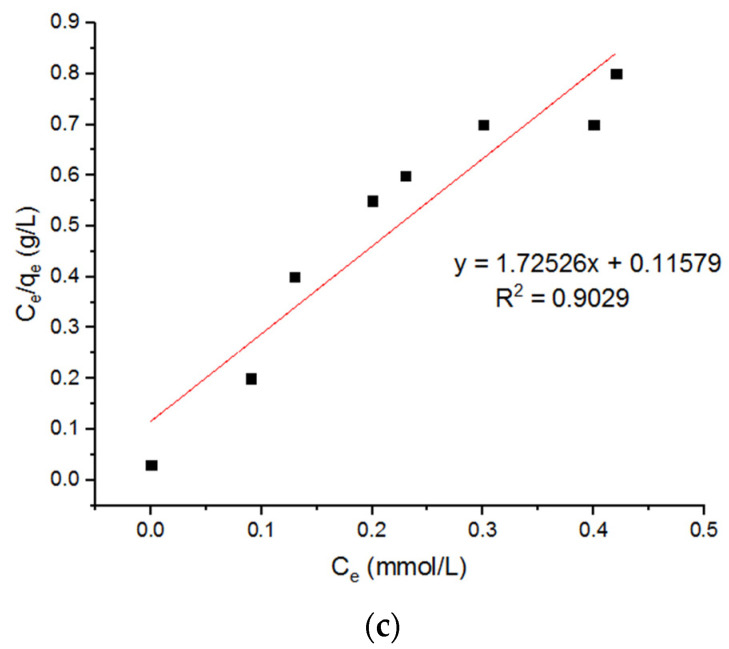
Langmuir plots for adsorption of Cu^2+^ ions onto MgFe_2_O_4_-NH_2_ nanoparticles pre-functionalization-calcined at (**a**) 500 °C, (**b**) 600 °C, and (**c**) 700 °C.

**Figure 14:**
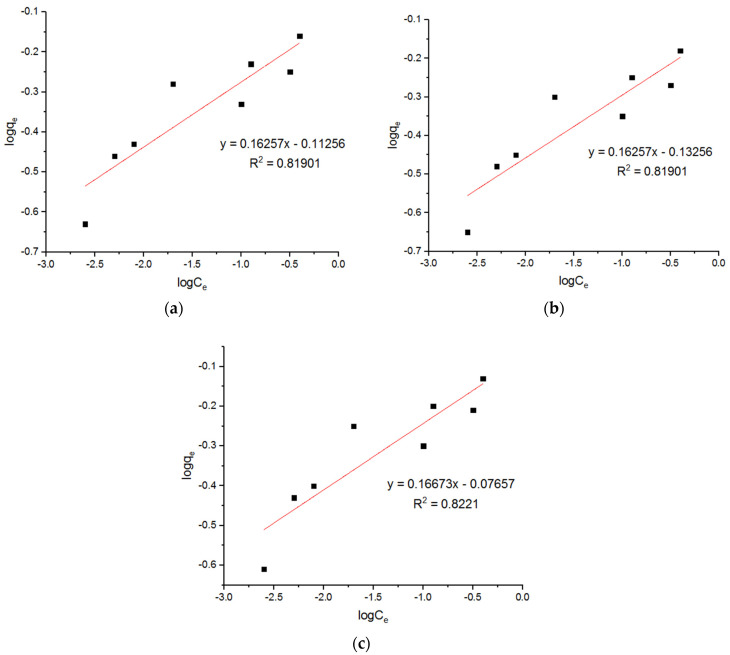
Freundlich plots for adsorption of Cu^2+^ onto MgFe_2_O_4_-NH_2_ nanoparticles pre-functionalization-calcined at (**a**) 500 °C, (**b**) 600 °C, and (**c**) 700 °C.

Three Langmuir plots for the adsorption of the Cu^2+^ ions onto the MgFe_2_O_4_-NH_2_ nanoparticles, calcined at 500 °C, 600 °C, and 700 °C, are shown in Figure 13, and the subsequent computed isotherm parameters are reported in Table 4. It is shown that the isotherm parameters at different calcination temperatures did not differ significantly. Three Freundlich plots for the adsorption of the Cu^2+^ ions onto the MgFe_2_O_4_-NH_2_ nanoparticles, pre-functionalization-calcined at 500 °C, 600 °C, and 700 °C, are shown in Figure 14, and the subsequent computed isotherm parameters are reported in Table 4, which indicates that the isotherm parameters at different calcination temperatures did not differ significantly. The results show that the Langmuir model has a higher R^2^ value than the Freundlich model, indicating that it fits better.

### 5.4. Isotherm Models for Pb^2+^ Adsorption onto MgFe_2_O_4_-NH_2_ Nanoparticles

**Figure 15:**
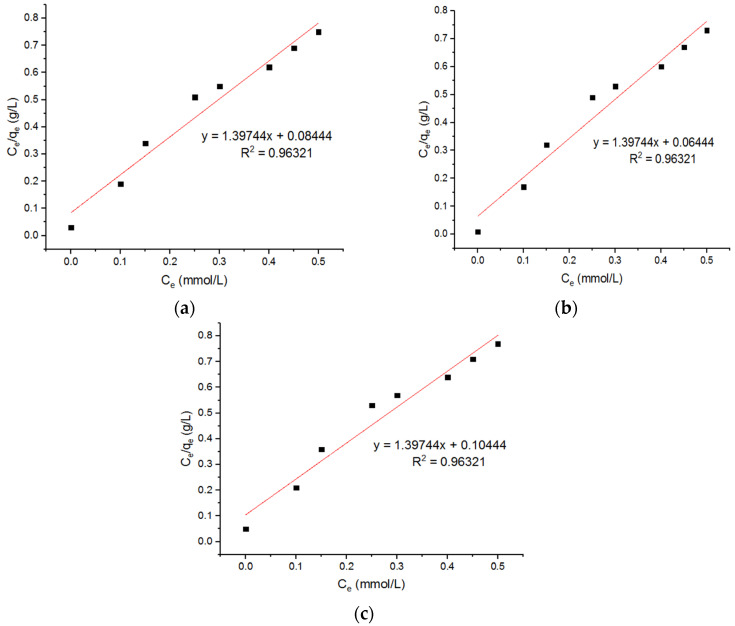
Langmuir plots for adsorption of Pb^2+^ onto MgFe_2_O_4_-NH_2_ NPs pre-functionalization-calcined at (**a**) 500 °C, (**b**) 600 °C, and (**c**) 700 °C.

**Figure 16:**
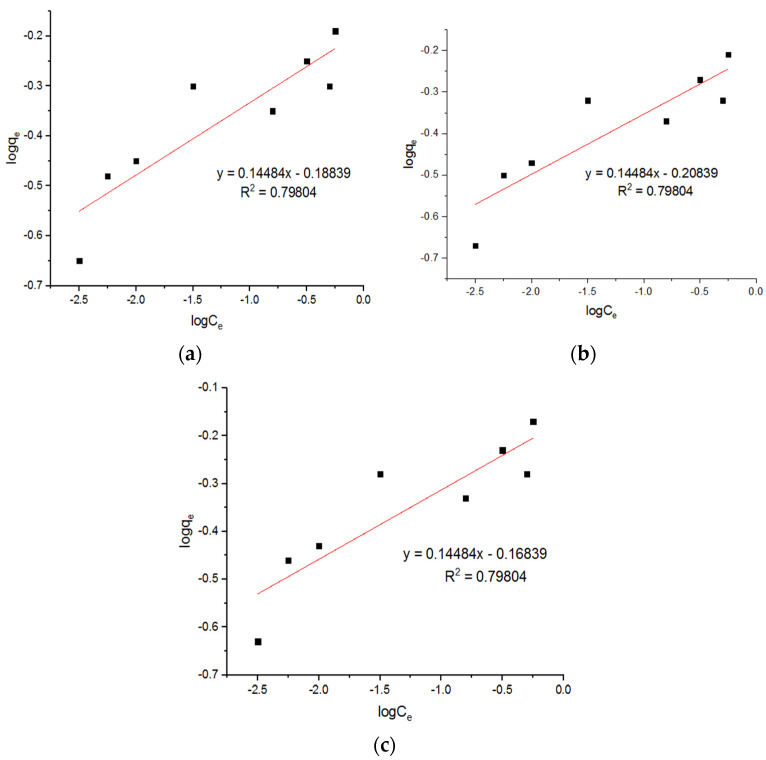
Freundlich plots for adsorption of Pb^2+^ onto MgFe_2_O_4_-NH_2_ nanoparticles pre-functionalization-calcined at (**a**) 500 °C, (**b**) 600 °C, and (**c**) 700 °C.

Tran et al. [24] reported that a Cu_0.5_Mg_0.5_Fe_2_O_4_ nano-adsorbent possessed a maximum capacity of 57.44 mg/g, as calculated from the Langmuir equation. The Langmuir plots for the adsorption of the Pb^2+^ ions onto the MgFe_2_O_4_-NH_2_ nanoparticles are shown in Figure 15, and the subsequent computed isotherm parameters are reported in Table 5. This shows that the isotherm parameters of the adsorbent, pre-functionalization-calcined at 500 °C and 700 °C, did not differ significantly. Three Freundlich plots for the adsorption of the Pb^2+^ onto the MgFe_2_O_4_-NH_2_ nanoparticles are shown in Figure 16, and subsequent computed isotherm parameters are reported in Table 5, which indicates that the isotherm parameters at different calcination temperatures are not significantly different.

### 5.5. Isotherm Models for Zn^2+^ Adsorption onto MgFe_2_O_4_-NH_2_ Nanoparticles

**Figure 17:**
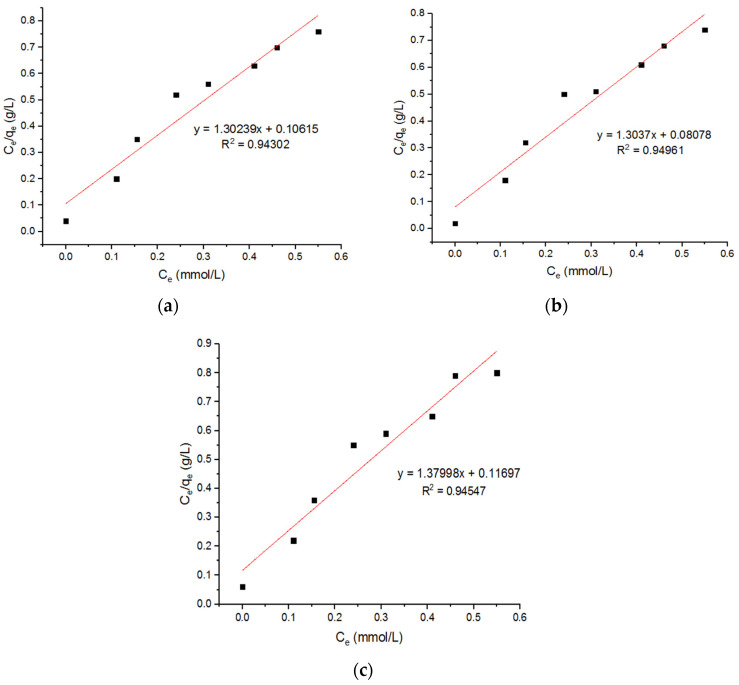
Langmuir plots for adsorption of Zn^2+^ ions onto MgFe_2_O_4_-NH_2_ nanoparticles pre-functionalization-calcined at (**a**) 500 °C, (**b**) 600 °C, and (**c**) 700 °C.

**Figure 18:**
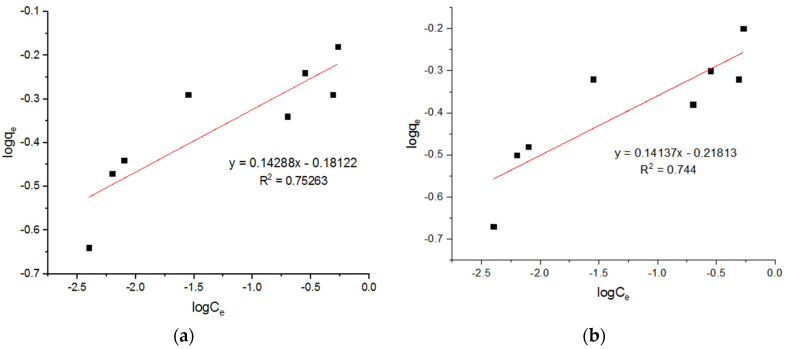

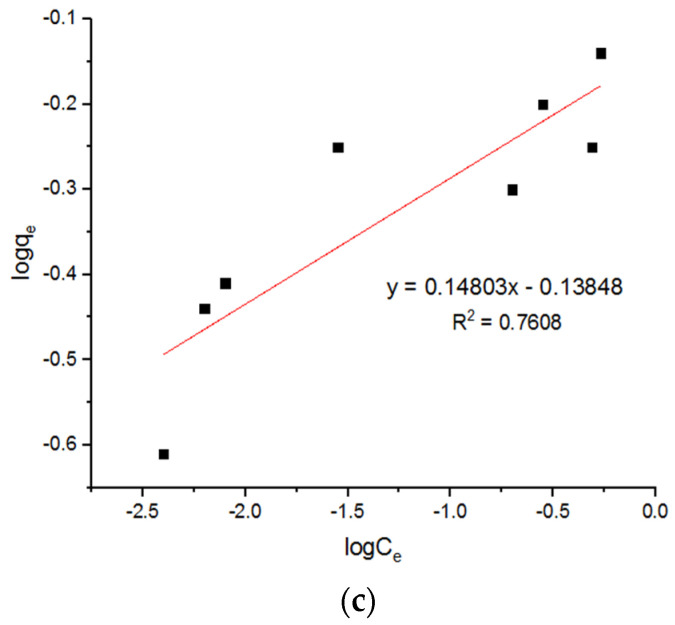
Freundlich plots for adsorption of Zn^2+^ ions onto MgFe_2_O_4_-NH_2_ nanoparticles pre-functionalization-calcined at (**a**) 500 °C, (**b**) 600 °C, and (**c**) 700 °C.

Three Langmuir plots for the adsorption of the Zn^2+^ ions onto the MgFe_2_O_4_-NH_2_ nanoparticles are shown in Figure 17, and the subsequent computed isotherm parameters are reported in Table 6. It is shown that the isotherm parameters at 500 °C and 700 °C did not change significantly. The Freundlich plots for the adsorption of the Zn^2+^ onto the MgFe_2_O_4_-NH_2_ nanoparticles, pre-functionalization-calcined at 500 °C, 600 °C, and 700 °C are shown in Figure 18, and the subsequent isotherm parameters are reported in Table 6. It is shown that the isotherm parameters at different calcination temperatures did not differ significantly. The results indicate that the value of R^2^ for the Langmuir isotherm was greater than the R^2^ value for the Freundlich adsorption isotherm. This confirms that the Langmuir isotherm is best fitted to heavy metal ion adsorption.

It can be seen that this adsorption process followed the pseudo-second-order model and the Langmuir model, as in the process reported by Nonkumwong et al. [15] and Fan et al. [26]. The present work shows that the pseudo-second-order model and Langmuir model were better fitted the adsorption of different metal ions. There was no difference between different metal ions when the adsorption was taken into account. Table 7 summarizes a comparison of the q_m_ values achieved in this study with those in other published literature.

The published literature shows greatly varying q_m_ values for removing heavy metal ions. Some studies revealed a high adsorption of Pb^2+^ ions on nano-adsorbents, while others showed a high adsorption of Cu^2+^ ions. Ren et al. [27] used magnetic porous ferrospinel MnFe_2_O_4_ to adsorb Pb^2+^ and Cu^2+^ ions. They reported q_m_ values of 69 and 37, respectively. Our study revealed q_m_ values of 145.04 and 55.7 for the Pb^2+^ and Cu^2+^ ions, respectively. Although MnFe_2_O_4_ and MnFe_2_O_4_ magnetic adsorbents fall in the same class of spinel ferrites, the high adsorbtion of metal ions by the latter is attributed to ethanolamine functionalization of the adsorbent. The presence of −NH_2_ groups on the surfaces MnFe_2_O_4_ nanoparticles enhances their adsorption capacity [28]. The heat treatment of the magnetic adsorbent also affects the level of functionalization and, consequently, the adsorption capacity. The number of −NH_2_ groups attached to the surface increases with an increase in calcination temperature and surface roughness.

Tran et al. [24] used a Mg_0.5_Cu_0.5_Fe_2_O_4_ composite magnetic adsorbent to remove Pb^2+^ ions. They revealed a q_m_ value of 57.7, which was significantly lower than the values reported by the current study. Duan et al. [29] used a Co_0.6_Fe_2.4_O_4_ magnetic adsorbent and reported q_m_ values for Pb^2+^ ions in the range of 44.58 to 70.22, depending on the adsorbent synthesis conditions. Tamez et al. [30] used magnetic Fe_3_O_4_ adsorbent for the removal of heavy metals from aqueous medium. They reported q_m_ values in the range of 47.62 to 166.67 and 19.61 to 37.04 for Pb^2+^ and Cu^2+^ ions, respectively. All these studies revealed much lower adsorption capacities than the current study. The low adsorption was attributed the pristine nature of the adsorbent. However, some other researchers composited the magnetic materials with non-magnetic materials for the construction of efficient adsorbents for heavy metals. For instance, Kalantari et al. [31] used Fe_3_O_4_/montmorillonite (Fe_3_O_4_/MMT NC) for the removal of Pb^2+^, Ni^2+^, and Cu^2+^ ions from aqueous solutions. They measured q_m_ values of 263.15 and 70.92 for the Pb^2+^ and Cu^2+^ ions, respectively. Mittal et al. [32] reported a poly(methyl methacrylate)-decorated alginate/Fe_3_O_4_ composite as a novel adsorbent for heavy metals. The adsorption data for Pb^2+^ and Cu^2+^ ions were analyzed using the Freundlich, Langmuir, Sips, and Temkin models. The experimental data were fitted best to the Freundlich model. The adsorption capacities were 62.5 mg/g and 35.71 mg/g for the Pb^2+^ and Cu^2+^ ions, respectively. Lasheen et al. [33] used magnetite chitosan (NMag–CS) films for the adsorption of heavy metals. They reported adsorption capacities of 114.9 mg/g and 123.4 mg/g for Pb^2+^ and Cu^2+^ ions, respectively. For all the metals, the adsorption kinetics followed a pseudo-second-order equation. The nanomagnetic materials showed better finding capacities for the heavy metal ions when combined with magnetic materials.

**Table 7:** A comparison of maximum adsorption capacities of different adsorbents.

Adsorbent	q_m_ (mg/g)		References
	Pb^2+^	Cu^2+^	
Magnetic porous ferrospinel MnFe_2_O_4_	69	37	[27]
EDTA-modified chitosan/SiO_2_/Fe_3_O_4_	12.5	44	[34]
Fe_3_O_4_@2,3-diaminophenol and formaldehyde nanorods	83	-	[35]
Chitosan-coated bentonite beads	-	12	[36]
Fe_3_O_4_-NH_2_ nanoparticles	40	-	[6]
Fe_3_O_4_@SiO_2_-NH_2_ nanoparticles	76	-	[37]
Mg_0.5_Cu_0.5_Fe_2_O_4_	57.7	-	[24]
Fe_3_O_4_/montmorillonite	263.15	70.92	[31]
PMMA-gft-Alg/Fe_3_O_4_	62.5	35.71	[32]
Co_0.6_Fe_2.4_O_4_	44.58 to 70.22	-	[29]
NMag–CS	114.9	123.4	[33]
Fe_3_O_4_	47.62 to 166.67	19.61 to 37.04	[30]
MgFe_2_O_4_-NH_2_ nanoparticles	145.04	55.7	Present work

## 6. Removal Efficiency

The removal efficiencies of different cations achieved by utilizing MgFe_2_O_4_-NH_2_ nanoparticles at different calcination temperatures are given in Table 8, which shows that the metal ions were largely removed by amine-functionalized nanoparticles pre-functionalization-calcined at 500 °C. Tan et al. [6] reported that about 98% of lead ions could be removed from tap water and wastewater by amino-functionalized Fe_3_O_4_ magnetic nanoparticles. The reported adsorbent cannot selectively capture different metal ions.

## 7. Conclusions

Magnesium ferrite (MgFe_2_O_4_) was successfully prepared by using the sol-gel method. The amine functionalization of the ferrite nanoparticles was carried out by using ethanolamine as a surface modifier. The kinetic and isotherm analysis of Pb^2+^, Cu^2+^, and Zn^2+^ adsorption on the surface of the MgFe_2_O_4_-NH_2_ was carried out after carefully optimizing the adsorption conditions. The synthesized materials had good removal efficiency and a fast adsorption rate. These results suggest a process for industrial wastewater treatment. The kinetic parameters and adsorption isotherms for the adsorption of the metal ions onto the amine-functionalized MgFe_2_O_4_ were obtained using the pseudo-first-order, pseudo-second-order, Langmuir, and Freundlich models. The pseudo-second-order and Langmuir models best described the adsorption kinetics and isotherms, implying strong chemisorption via the formation of coordinative bonds between the amine groups and metal ions. The Langmuir equation revealed the highest adsorption capacity of 0.7 mmol/g for the amine-functionalized MgFe_2_O_4_ nano-adsorbent. The calcination temperature also had an effect on the adsorption capacity of the nano-adsorbent. The MgFe_2_O_4_ sample, which was calcined at 500 °C, removed the most Pb^2+^ (73%), Cu^2+^ (59%), and Zn^2+^ (62%) from the effluent.

## Figures and Tables

**Table 1:** Pseudo-first- and second-order-based adsorption rate constant for Cu^+2^ ions.

Adsorption of Cu^+2^ onto MgFe_2_O_4_-NH_2_ NPs (First-Order Model)								
k_1_ (min^−1^)	q_e_ (mg/g)	R^2^	k_1_ (min^−1^)	q_e_ (mg/g)	R^2^	k_1_ (min^−1^)	q_e_ (mg/g)	R^2^
0.036	6.89	0.8242	0.032	6.55	0.79131	0.037	6.61	0.82238
Adsorption of Cu^+2^ onto MgFe_2_O_4_-NH_2_ NPs (Second-Order Model)								
500 °C			600 °C			700 °C		
k_2_ (g/mg. min)	q_e_ (mg/g)	R^2^	k_2_ (g/mg. min)	q_e_ (mg/g)	R^2^	k_2_ (g/mg. min)	q_e_ (mg/g)	R^2^
−0.291	39.68	0.95623	−0.018	50.20	0.95042	−0.136	39.38	0.96127

**Table 2:** Pseudo-first- and second-order-based adsorption rate constant.

Adsorption of Pb^2+^ onto MgFe_2_O_4_-NH_2_ NPs (Pseudo-First-Order Model)								
500 °C			600 °C			700 °C		
k_1_ (min^−1^)	q_e_ (mg/g)	R^2^	k_1_ (min^−1^)	q_e_ (mg/g)	R^2^	k_1_ (min^−1^)	q_e_ (mg/g)	R^2^
0.042	7.24	0.86184	0.0425	7.50	0.86836	0.043	7.23	0.87433
Adsorption of Pb^2+^ onto MgFe_2_O_4_-NH_2_ NPs (Pseudo-Second-Order Model)								
500 °C			600 °C			700 °C		
k_2_ (g/mg. min)	q_e_ (mg/g)	R^2^	k_2_ (g/mg. min)	q_e_ (mg/g)	R^2^	k_2_ (g/mg. min)	q_e_ (mg/g)	R^2^
−0.0136	35.98	0.9906	−0.011	39.2	0.98648	−0.096	33.68	0.98048

**Table 3:** Adsorption rate constant calculated from pseudo models.

Adsorption of Zn^+2^ onto MgFe_2_O_4_-NH_2_ NPs (Pseudo-Firstst-Order Model)								
500 °C			600 °C			700 °C		
k_1_ (min^−1^)	q_e_ (mg/g)	R^2^	k_1_ (min^−1^)	q_e_ (mg/g)	R^2^	k_1_ (min^−1^)	q_e_ (mg/g)	R^2^
0.039	7.106	0.83718	0.037	7	0.83735	0.0395	6.79	0.83718
Adsorption of Zn^2+^ onto MgFe_2_O_4_-NH_2_ NPs (Pseudo-Second-Order Model)								
500 °C			600 °C			700 °C		
k_2_ (g/mg. min)	q_e_ (mg/g)	R^2^	k_2_ (g/mg. min)	q_e_ (mg/g)	R^2^	k_2_ (g/mg. min)	q_e_ (mg/g)	R^2^
0.141	35.42	0.98555	−0.0173	38.95	0.91528	0.01	34.98	0.97874

**Table 4:** Isotherm parameters calculated from Langmuir and Freundlich model.

Langmuir Model for Adsorption of Cu^2+^ onto MgFe_2_O_4_-NH_2_ Nanoparticles								
500 °C			600 °C			700 °C		
q_m_ (mmol/g)	K_L_ (L/mmol)	R^2^	q_m_ (mmol/g)	K_L_ (L/mmol)	R^2^	q_m_ (mmol/g)	K_L_ (L/mmol)	R^2^
0.657	16.324	0.9019	0.877	17.84	0.92035	0.579	14.92	0.9029
Freundlich Model for Adsorption of Cu^2+^ onto MgFe_2_O_4_-NH_2_ Nanoparticles								
500 °C			600 °C			700 °C		
K_F_ (mmol^1(1/n)^L^1/n^/g)	n	R^2^	K_F_ (mmol^1(1/n)^L^1/n^/g)	n	R^2^	K_F_ (mmol^1(1/n)^L^1/n^/g)	n	R^2^
0.77	6.15	0.8190	0.73	6.15	0.8190	0.84	5.9	0.8221

**Table 5:** Isotherm parameters calculated from Langmuir model.

Langmuir Model for Adsorption of Pb^2+^ Ions onto MgFe_2_O_4_-NH_2_ Nanoparticles Pre-Functionalization Thermally Treated at 500 °C, 600 °C, and 700 °C.								
500 °C			600 °C			700 °C		
q_m_ (mmol/g)	K_L_ (L/mmol)	R^2^	q_m_ (mmol/g)	K_L_ (L/mmol)	R^2^	q_m_ (mmol/g)	K_L_ (L/mmol)	R^2^
0.715	16.56	0.96321	0.7155	21.69	0.96321	0.7156	13.38	0.96321
Freundlich Model for Adsorption of Pb^2+^ Ions onto MgFe_2_O_4_-NH_2_ Nanoparticles Pre-Functionalization Thermally Treated at 500 °C, 600 °C, and 700 °C.								
500 °C			600 °C			700 °C		
K_F_ (mmol^1(1/n)^L^1/n^/g)	n	R^2^	K_F_ (mmol^1(1/n)^L^1/n^/g)	n	R^2^	K_F_ (mmol^1(1/n)^L^1/n^/g)	n	R^2^
0.648	6.9	0.79804	0.61	6.90	0.7984	0.67	6.9	0.79804

**Table 6:** Isotherm parameters calculated from Langmuir model.

Langmuir Model for Adsorption of Zn^2+^ Ions onto MgFe_2_O_4_-NH_2_ Nanoparticles Pre-Functionalization Thermally Treated at 500 °C, 600 °C, and 700 °C.								
500 °C			600 °C			700 °C		
q_m_ (mmol/g)	K_L_ (L/mmol)	R^2^	q_m_ (mmol/g)	K_L_ (L/mmol)	R^2^	q_m_ (mmol/g)	K_L_ (L/mmol)	R^2^
0.768	12.27	0.94302	0.767	16.14	0.94961	0.724	11.80	0.94547
Freundlich Model for Adsorption of Zn^2+^ Ions onto MgFe_2_O_4_-NH_2_ Nanoparticles Pre-Functionalization Thermally Treated at 500 °C, 600 °C, and 700 °C.								
500 °C			600 °C			700 °C		
K_F_ (mmol^1(1/n)^L^1/n^/g)	N	R^2^	K_F_ (mmol^1(1/n)^L^1/n^/g)	n	R^2^	K_F_ (mmol^1(1/n)^L^1/n^/g)	n	R^2^
0.658	6.99	0.75263	0.605	7.07	0.744	0.727	6.75	0.7608

**Table 8:** Metal ion removal efficiency of adsorbent pre-functionalization thermally treated at 500 °C, 600 °C, and 700 °C.

Metal Ions	Removal Efficiency at 500 °C (%)	Removal Efficiency at 600 °C (%)	Removal Efficiency at 700 °C (%)
Pb^2+^	73	58	55
Cu^2+^	59	58	56
Zn^2+^	62	58	56

## Data Availability

Not applicable.
